# Increases in Acute Phase Reactants in a Patient with Scurvy Despite No Inflammation: Review of Literature

**DOI:** 10.7759/cureus.5608

**Published:** 2019-09-09

**Authors:** Kaori Kimura, Yasuji Inamo

**Affiliations:** 1 Pediatrics, Nihon University School of Medicine, Tokyo, JPN; 2 Pediatrics, Nihon Uniiversity School of Medicine, Tokyo, JPN

**Keywords:** scurvy, vitamin c, c-reactive protein, acute phase reactants, arthritis

## Abstract

So far, little attention has been paid to the increase in acute phase reactants (APRs) in patients with scurvy. We report that elevated levels of C-reactive protein (CRP), erythrocyte sedimentation rate (ESR), and serum amyloid A were shown in a pediatric patient with scurvy despite the absence of inflammation. These peculiar findings are important to discriminate scurvy from other rheumatic diseases.

## Introduction

An increase in acute phase reactants (APRs) has not been observed in patients with scurvy. In fact, there are few cases of scurvy with descriptive comments on APRs [1]. Pediatric rheumatologists usually measure APRs to evaluate the level of inflammation in patients with leg pain or a limp. Because patients with scurvy complain of leg pain or a limp, it is necessary to discriminate it from other rheumatological diseases. Thus, we aim to discuss why elevated APRs, including C-reactive protein (CRP), in scurvy is an important clinical problem.

## Case presentation

A 4-year-old male with autism was referred to our hospital because of severe leg pain and gingival bleeding. His laboratory results showed a CRP level of 2.21 mg/dL (reference range <0.05 mg/dL), a serum amyloid A protein level of 544 μg/ml (reference range <8 μg/ml), and an erythrocyte sedimentation rate (ESR) of 94 mm/hour (reference range 5-15 mm/hour). His leg pain did not subside with acetaminophen. His physical symptoms and APRs improved quickly on the administration of vitamin C after a diagnosis of scurvy.

## Discussion

Although several reports have described the levels of elevated CRP, ESR, and serum amyloid A, they have not garnered enough attention (Table 1) [1-7]. The peculiar findings could be misleading as an inflammatory reaction. But, an increase in acute phase reactants (APRs) in scurvy should be recognized as a common phenomenon.

**Table 1:** Results of laboratory tests on CRP and ESR in patients with scurvy *Described as almost normal on the article. **ND: not described CRP; C-reactive protein, ESR; erythrocyte sedimentation rate

Case no. (from references of citation)	Age (year)	CRP (mg/L)	ESR (mm/hour)	Reference
sex, (reference range)				
1	26	4	41	[1]
	male	(ND**)	(ND**)	
2	22	13.4	101	[1]
	male	(ND**)	(ND**)	
3	74	ND**	41	[1]
	female		(ND**)	
4	6	31.1	103	[2]
	male	(0.1-1)	(2-34)	
5	9	24.5	59	[3]
	male	(< 8.0)	(0-17)	
6	12	almost normal*	44	[4]
	male		(0-15)	
7	9	1.3	23	[5]
	male	(<0.5)	(0-20)	
8	5	5.81	44	[6]
	male	(<0.3)	(0-15)	
9	10	10.9	ND**	[7]
	male	(<1)		
Our case	4	22.1	94	
	male	(<0.5)	(5-15 )	

It is difficult to interpret elevations in APRs that are caused by local bone lesions in scurvy, which, by radiology, are reflected by increases in zones of provisional calcification in the margins of the growth plate. Conversely, CRP does not increase in vitamin D deficiency rickets, which appears in radiological findings as a failure of mineralization in such zones. The levels of APRs fall after the administration of vitamin C, but anti-inflammatory drugs do not have the same effect. Therefore, it is difficult to conclude that inflammation due to scurvy induces a rise in APRs.

It is well-known that plasma vitamin C is inversely related to CRP [8]. Both, the low levels of serum vitamin C and the levels of elevated CRP, may participate in atherothrombosis and ischemic heart disease. However, the levels of CRP in scurvy are higher than those in atherosclerosis. Only CRP, apart from the other APRs, increases in atherosclerosis. The source of CRP in scurvy might be different from the vascular endothelial site in atherosclerosis. Vitamin C deficient rats without inflammatory stimuli experience an increase in APRs, which are produced in the liver [9-10]. Although the mechanism by which APRs are elevated in scurvy remains unknown, these rats can provide vital clues for elucidating it.

We believe that a deficiency of serum vitamin C itself can generate APRs, to resemble an aspect of inflammatory biomarkers. The specific findings are important to discriminate scurvy from any other rheumatic diseases with an inflammatory reaction.

## Conclusions

We conclude that the depletion of vitamin C (i.e., scurvy) leads to the production of APRs in the liver, the laboratory results for which resemble acute inflammation despite a lack of inflammation.
